# Clinical Management and Treatment Guidelines for Intraforaminal Lumbar Synovial Cyst Presentation: Report of Two Cases

**DOI:** 10.7759/cureus.77733

**Published:** 2025-01-20

**Authors:** Aasim Z Hawa, Lancelot Benn, Addisu Mesfin

**Affiliations:** 1 College of Medicine, University of Rochester School of Medicine and Dentistry, Rochester, USA; 2 Orthopaedic Surgery, MedStar Washington Hospital Center, Washington, DC, USA

**Keywords:** decompression, fusion, intraforaminal, laminectomy, lumbar synovial cysts, mis techniques, orthopedic surgery

## Abstract

We report two cases of unusually located foraminal lumbar synovial cysts: a 46-year-old female and a 59-year-old male, both of whom experienced pain and/or associated numbness. One patient underwent decompressive laminectomy and cyst resection, while the other required concomitant interbody fusion. All patients reported symptom resolution with no recurrence of the cysts. The foraminal location for lumbar synovial cysts is relatively uncommon and lacks conclusive treatment guidelines. Notably, the necessity for arthrodesis in addition to decompression remains controversial. However, these cases suggest that surgical resection of these cysts can be successful.

## Introduction

Lumbar synovial cysts (LSCs) are non-cancerous, fluid-filled structures typically occurring at the Lumbar Segment 4 - Lumbar Segment 5 (L4-L5 level) [1]. These cysts can contribute to spinal stenosis and may be associated with symptoms including radiculopathy, claudication, and cauda equina syndrome [1,2]. Although the precise etiology of LSCs is unclear, cyst formation frequently arises from degenerated facet joints, which can contribute to significant spondylostenosis [3]. When LSCs are relatively small, they can often be clinically dormant and found incidentally [3]. However, the epidural growth of these structures can provoke spinal compression, manifesting through the aforementioned symptoms [1].

LSCs were first described as causing spinal nerve compression by Vosschulte K and Borger G [2,4]. CT and MRI are two imaging modalities frequently used in the diagnosis and characterization of LSCs [1,2]. MRI has been particularly effective as a diagnostic tool; such images reveal the complexity of the lesion in addition to its spatial relation to the dural sac and other anatomical features [1,3]. Although LSCs have been treated effectively using conservative treatment modalities such as percutaneous aspiration and corticosteroid injection, surgical excision and decompression have been the preferred treatments, particularly for advanced cases [1]. More recently, controversy has emerged regarding whether arthrodesis, in addition to decompression, may be a more effective treatment modality than decompression alone [5]. Some argue that because LSCs are frequently associated with spinal instability, concomitant surgical fusion may be valuable [6].

A broad range of criteria for the successful treatment of LSCs exists within the literature, including the Oswestry Disability Index (ODI), peri- and post-operative complications, cyst recurrence, and rate of reoperation [2,3,6]. The latter two indicators have been recognized as particularly relevant clinically [5,6]. Following traditional decompressive techniques, it has been estimated that cyst recurrence can range between 10-15% [5-7]. Conversely, a recent study following 32 patients treated with concomitant decompression and fusion indicated no postoperative cyst recurrence [5]. Additional literature reviews and independent case reports corroborate such findings of near-zero cyst recurrence following this combination surgery as a treatment modality for LSCs [8,9]. However, other studies have found no significant improvement in cyst recurrence following decompression-fusion operations [10].

Synovial cysts can also present in unusual locations within and near the foraminal region, though such cases are quite uncommon, with only 15 published in the literature to our knowledge [11-20]. The necessity for fusion remains controversial in the treatment of foraminal LSCs [12]. In addition to providing insight into this debate, contributing details of the following cases to the literature can be valuable in guiding future therapies and treatment decisions in this relatively under-discussed field.

Patients with foraminal LSCs may present with radicular pain, numbness, tingling, or weakness that typically follows a dermatomal pattern. In these patients, lateral flexion or extension of the spine can lead to further compression of the nerve root, which can worsen symptoms. Conversely, patients with central canal LSCs may experience neurogenic claudication, characterized by pain, weakness, or a feeling of heaviness in the legs while walking or standing, which is often alleviated by sitting or bending the spine. Extension of the spine may aggravate central stenosis, whereas flexion tends to open the canal slightly, offering symptomatic relief. The surgical options for these patients include decompressive laminectomy and cyst resection, with or without concomitant interbody fusion.

Informed consent was obtained from the two patients involved in this case report according to the IRB guidelines of the University of Rochester Medical Center. The patients consented to submitting information concerning their cases for publication.

## Case presentation

Case 1

**Figure 1:**
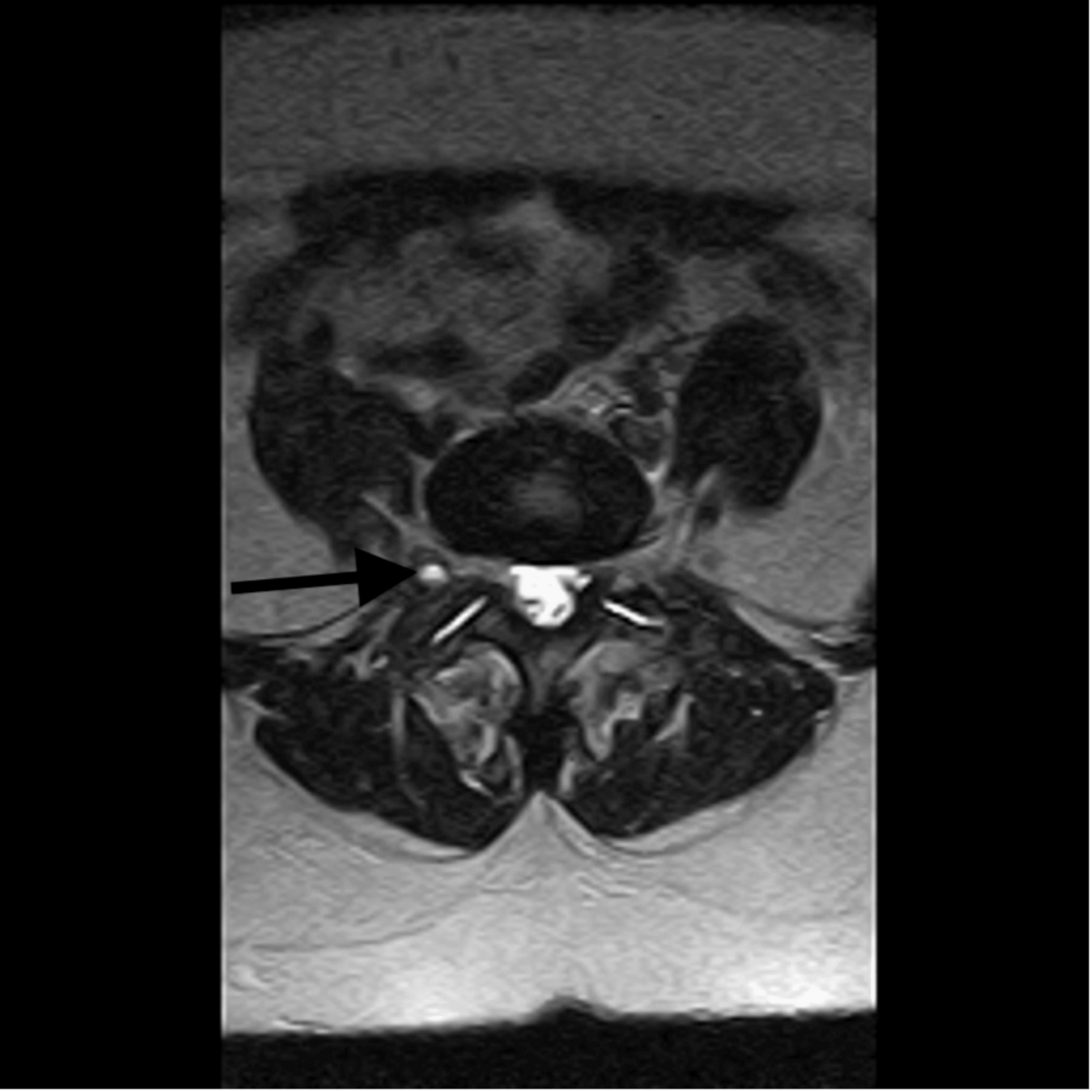
Axial MRI view of the lumbar spine for Case 1. Black arrow indicates a synovial cyst at L5-S1 with adjacent fluid causing instability. L5: Lumbar Segment 5; S1: Sacral Segment 1.

**Figure 2:**
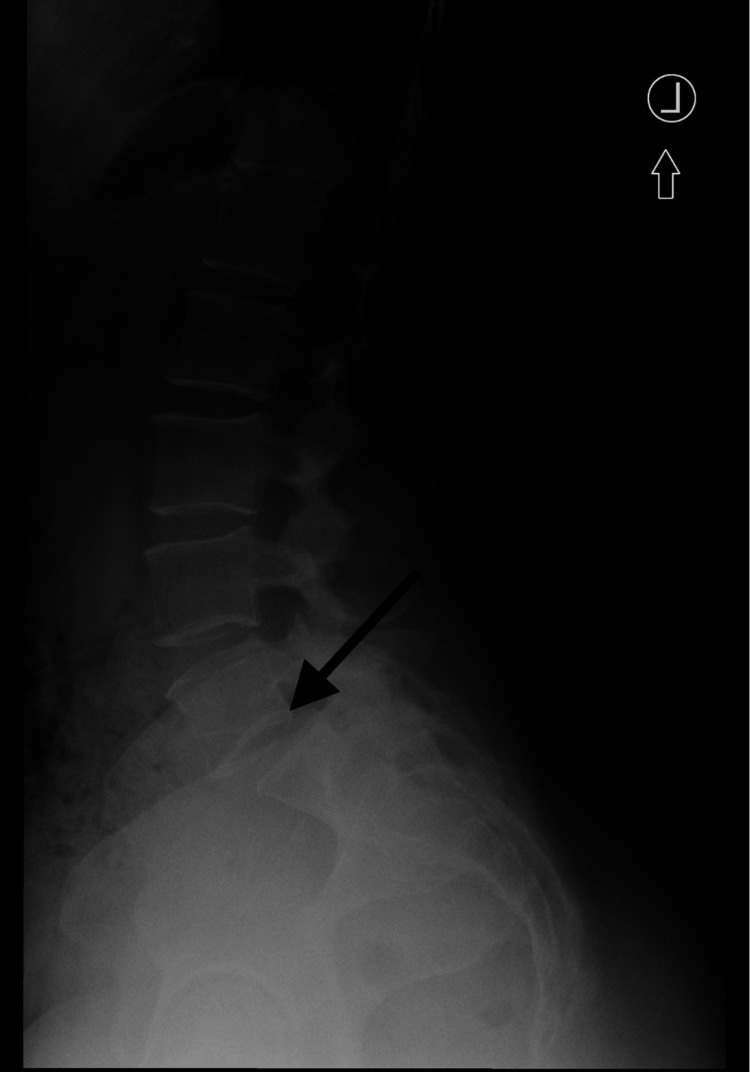
Lateral radiograph of the lumbar spine for Case 1. The black arrow indicates lumbar spondylolisthesis at L5-S1. L5: Lumbar Segment 5; S1: Sacral Segment 1.

A 46-year-old female with a prolonged history of right leg pain was diagnosed with a right intraforaminal synovial cyst at L5-S1 (Lumbar Segment 5-Sacral Segment 1), as indicated by an MRI of the lumbar spine (Figure 1). Further, radiography demonstrated arthropathy at L5-S1 facets, in addition to degenerative spondylolisthesis (Figure 2). The MRI specifically noted the location of the LSC. Nonoperative management was unsuccessful; thus, the patient was scheduled for L5-S1 posterior spinal fusion, including laminectomy and cyst resection.

**Figure 3:**
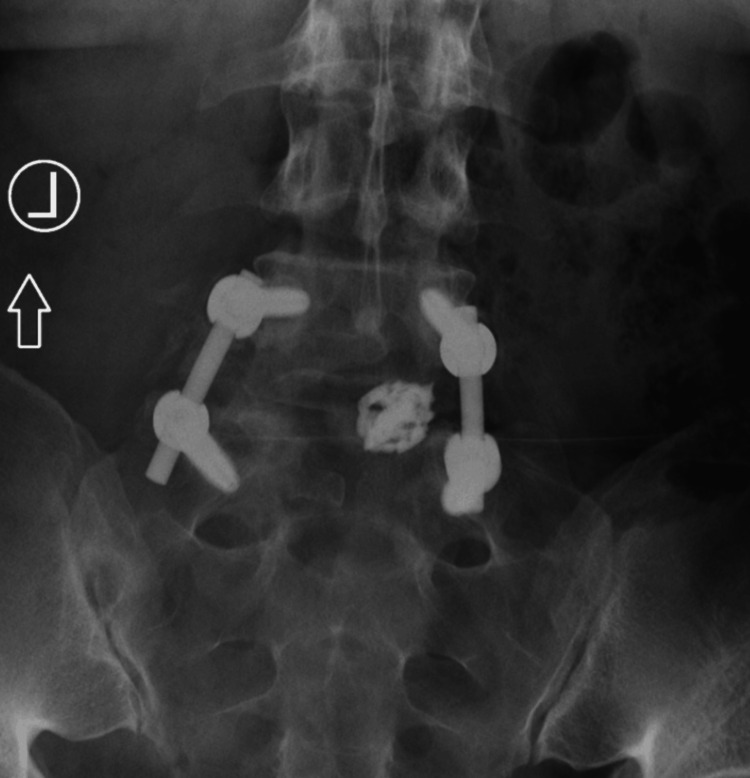
Anteroposterior X-ray of the lumbar spine for Case 1 following the procedure.

**Figure 4:**
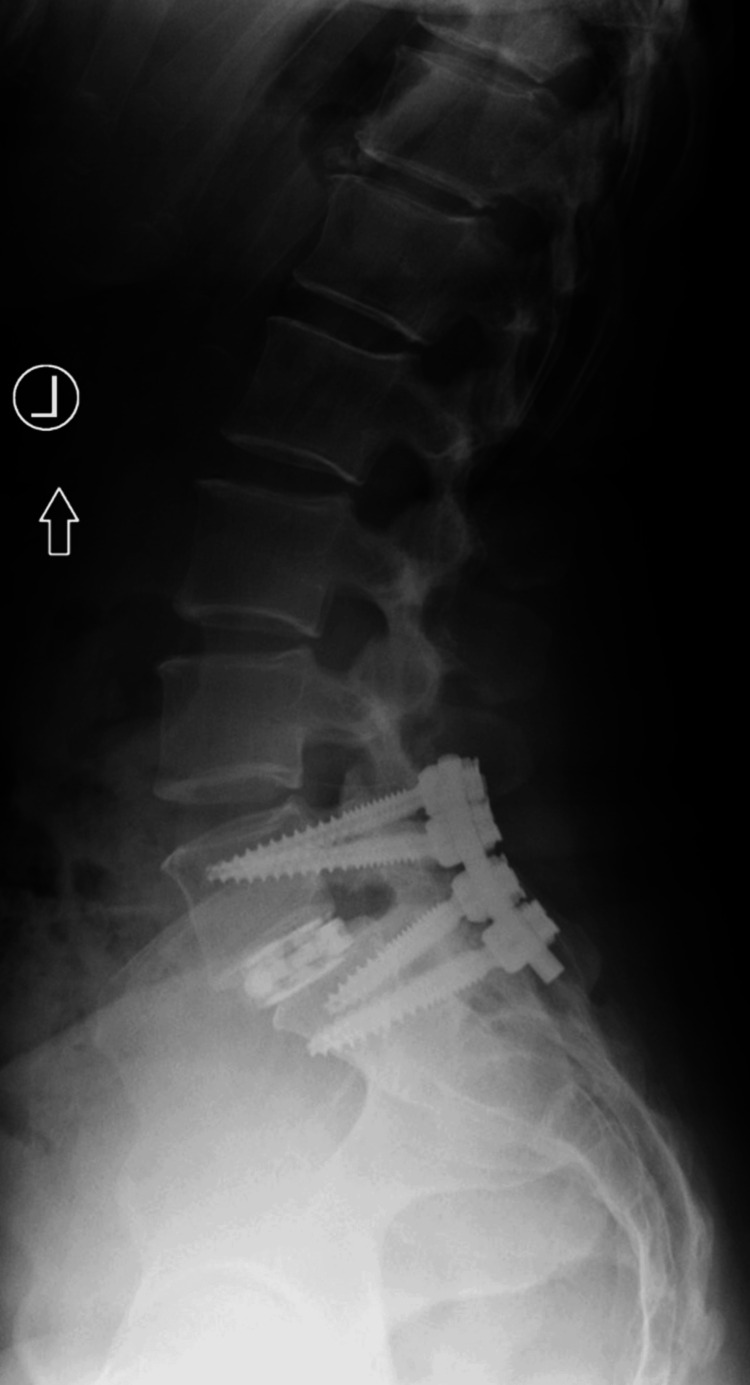
Post-operative lateral radiograph for Case 1.

Posterior spinal fusion with interbody fusion, laminectomy, and removal of the extradural lesion was performed successfully. Post-operative imaging showed the implantation of a titanium disc to assist with fusion (Figures 3-4). Additionally, pathology confirmed the resected soft-tissue specimen as representative of a synovial cyst. At the follow-up visit 36 months post-operation, the patient reported symptom resolution.

Case 2

**Figure 5:**
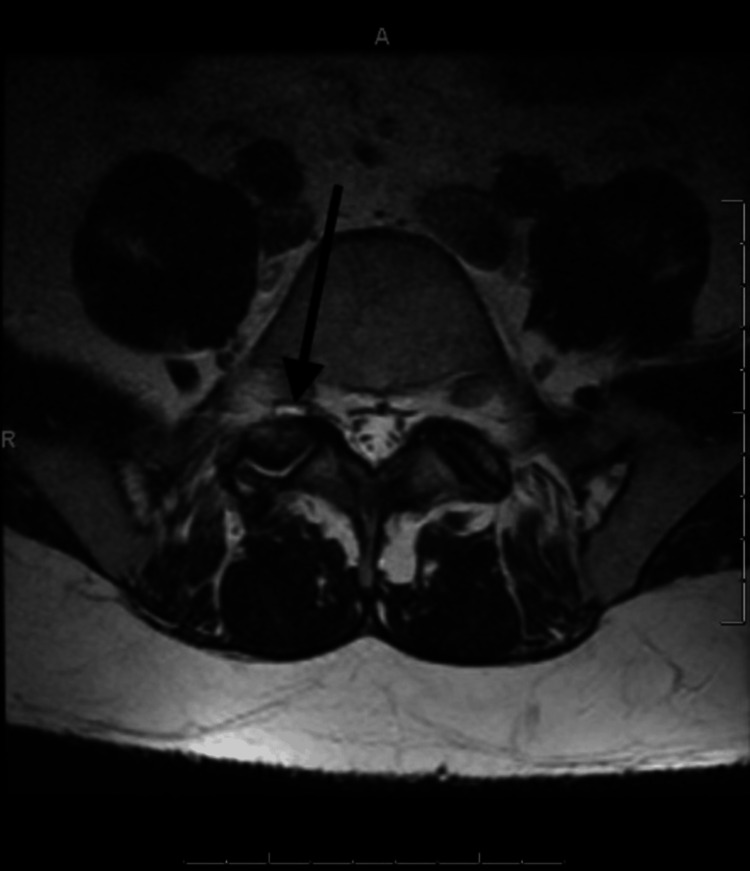
Axial MRI view of the lumbar spine for Case 2. Black arrow indicates a subtle synovial cyst at L5-S1. L5: Lumbar Segment 5; S1: Sacral Segment 1.

**Figure 6:**
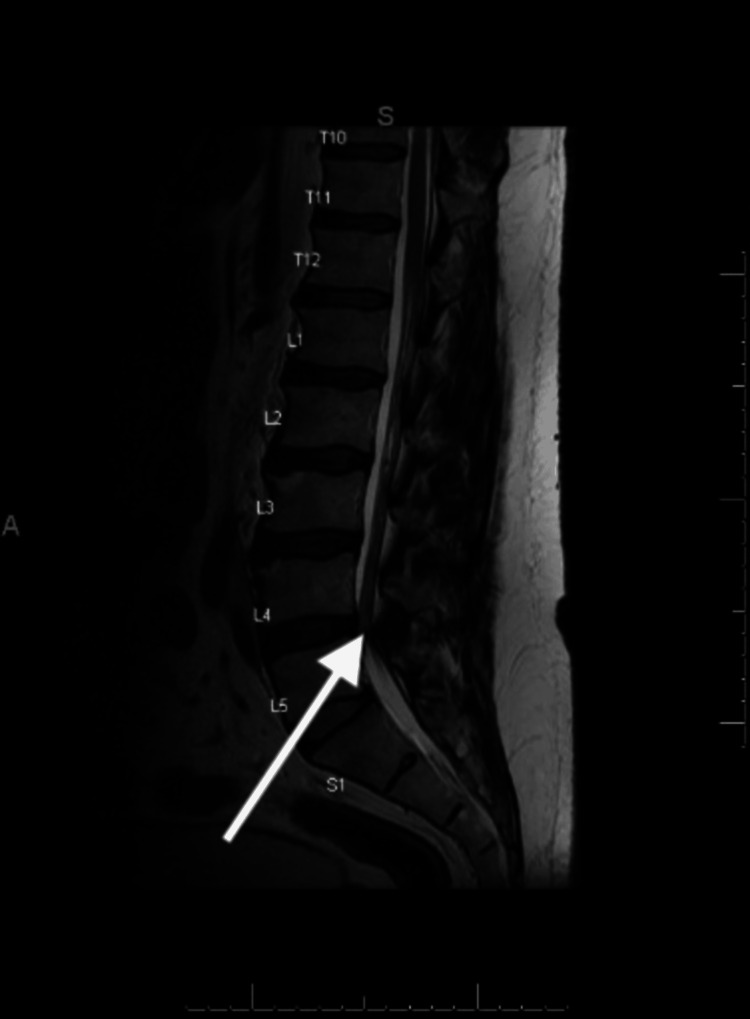
Sagittal MRI view of the lumbar spine for Case 2, with a white arrow pointing to spinal canal narrowing.

A 59-year-old male presented with bilateral lower extremity numbness but minimal pain. Imaging revealed a subtle right intraforaminal cyst at L5-S1 (Figure 5), in addition to central canal stenosis at L4-L5 (Figure 6). Surgical intervention, consisting of L4-L5 laminectomy, L5-S1 laminectomy, and synovial cyst resection, was planned.

These procedures were successful, with effective decompression. Notably, interbody fusion was not necessary. Histological analysis confirmed the resected specimen as consistent with LSC composition. At the 6-month post-operative follow-up, the patient reported improvement in numbness. Three years later, the patient reported symptom resolution.

**Figure 7:**
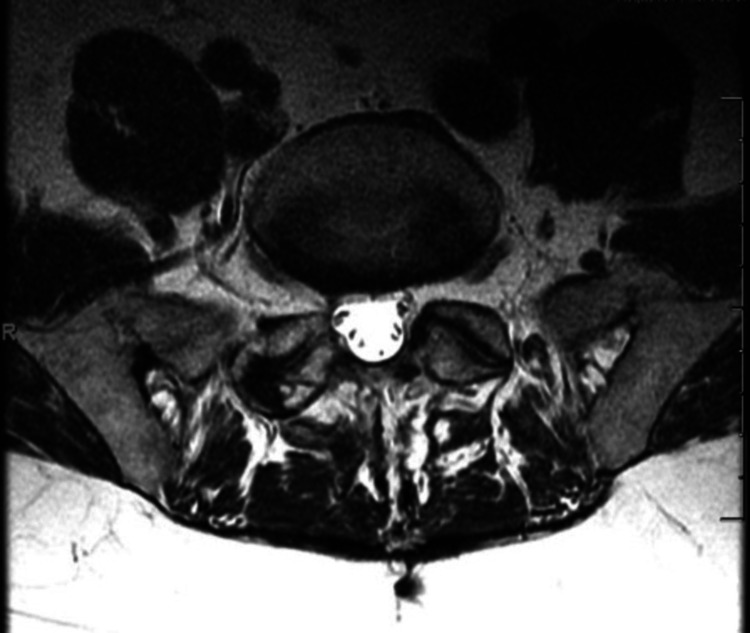
Axial view of the lumbar spine from a three-year post-operative MRI for Case 2.

**Figure 8:**
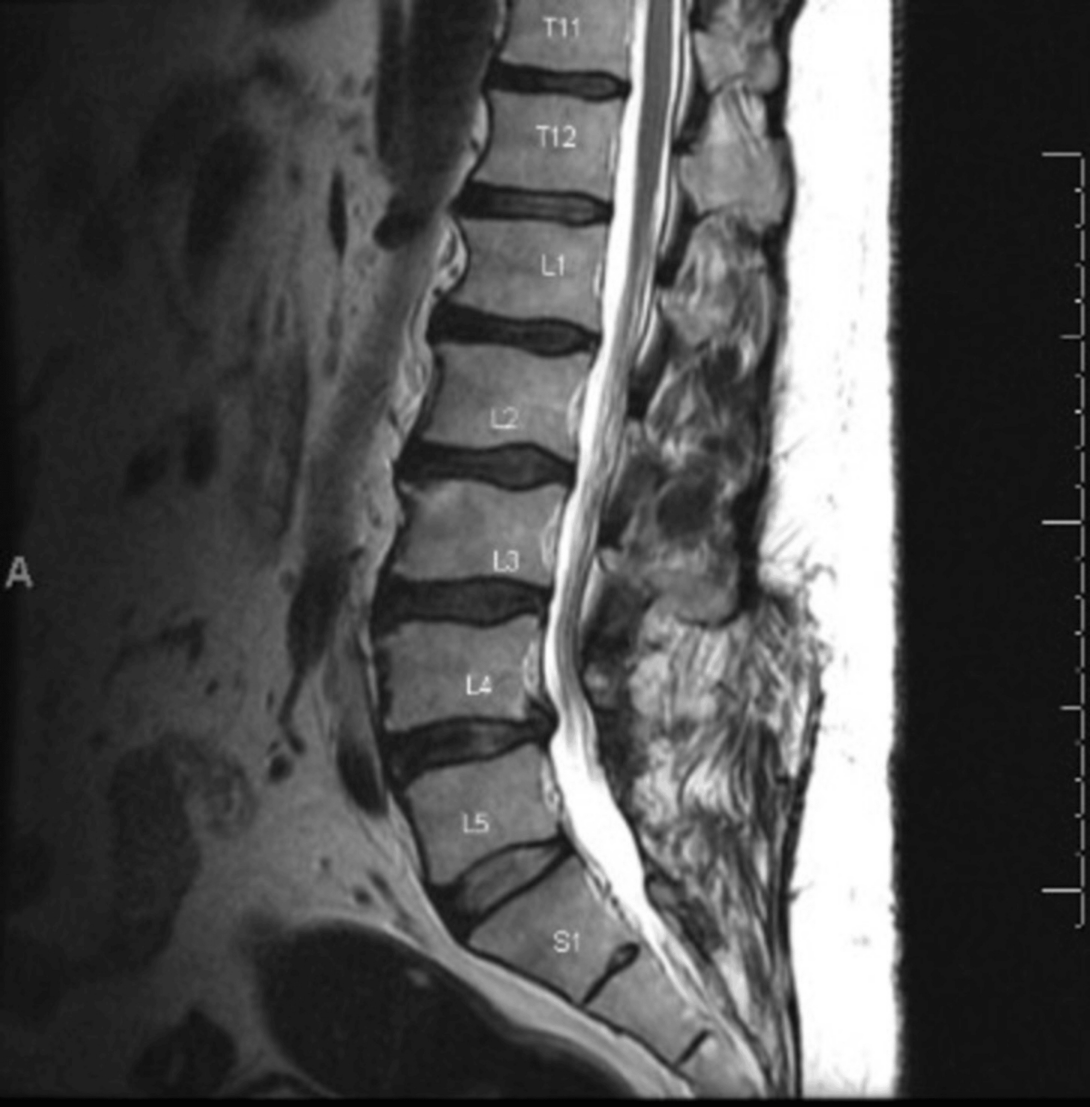
Sagittal MRI view of the lumbar spine for Case 2 following surgical decompression.

Furthermore, an MRI of the lumbar spine performed 40 months post-operation indicated a widening of the lumbar spinal canal in addition to the absence of the cyst specimen (Figures 7-8). Notably, the patient was diagnosed with unrelated bursitis eight months following this second follow-up visit. The patient reported symptom resolution at the latest follow-up, 40 months post-operation.

## Discussion

The foraminal location for LSCs is relatively uncommon, with the majority of LSCs confined to the spinal canal [12,13]. Such specimens can cause spinal nerve compression and are frequently detected using CT or MRI following symptom onset [2]. Although continuity between the cyst and the synovium of the adjacent facet joint is not always observed, it is generally understood that LSCs are joint-derived [15,16,18].

To our knowledge, 15 cases of such unusually located LSCs have been reported (Table 1) [11-20]. Notably, fusion was only performed in two cases [19]. Even without concomitant arthrodesis, all studies with follow-ups indicated symptom resolution or improvement, without cyst recurrence (Table 1). In the two cases reported herein, all patients reported symptom relief, with only one undergoing interbody fusion.

**Table 1:** Reported cases of foraminal lumbar synovial cysts (LSCs). N/A: Not Applicable; L3: Lumbar Segment 3, L4: Lumbar Segment 4, L5: Lumbar Segment 5; S1: Sacral Segment 1.

Reference	Age	Sex	Chief Presenting Complaint	Post-Imaging Diagnosis	Surgical Technique	Follow-up	Follow-up Period	Additional Comments
Kemaloğlu S et al. [11]	26	Male	3-month history of left leg pain	Left L4-5 cyst	Hemipartial laminectomy of L4 and L5, and cyst resection	No cyst recurrence	2 months	
Salmon BL et al. [17]	62	Male	Acute inaugural back pain and left sciatica	Left L5 cyst	Cyst resection	No cyst or symptom recurrence	1 year	Presence of drop foot
Maupin WB et al. [14]	57	Female	6-month history of left hip, buttock, and leg pain	Left L5-S1 cyst	Cyst resection	Not mentioned	N/A	
Spinner RJ et al. [18]	N/A	N/A	N/A	Right L5 cyst	Total facetectomy, and cyst resection	N/A	N/A	All cases grouped and discussed in association without patient specifics
	N/A	N/A	N/A	L5-S1 cyst	Total facetectomy, and cyst resection	N/A	N/A	
	N/A	N/A	N/A	L5 cyst	Total facetectomy, and cyst resection	N/A	N/A	
	N/A	N/A	N/A	N/A	Partial facetectomy, and cyst resection	N/A	N/A	
Phuong LK et al. [16]	42	Female	3-month history of sharp pain in lower right extremity	Right L5-S1 cyst	Total facetectomy, and cyst resection	Symptom resolution	Not mentioned	
	66	Female	3-month history of worsening pain in lower right extremity	Right L5-S1 cyst with effacement of L5 nerve	Total facetectomy, and cyst resection	Symptom resolution	Not mentioned	
Oertel MF et al. [15]	56	Female	Lower back pain	Left L3-L4 cyst with compression of L3 nerve	Partial hemilaminectomy and flavectomy	No symptom recurrence	1 year	Microsurgical approach
Kim JU et al. [12]	52	Female	Dull pain over bilateral buttocks	Right L4-5 cyst with compression of L4 nerve	Micro-decompression, and cyst resection	No cyst or symptom recurrence	6 months	Presence of drop foot
Manabe M et al. [13]	54	Female	15-month history of worsening pain in lower right extremity	Right L5-S1 cyst	L5 decompression, recapping right isthmectomy and facetectomy, and cyst resection	No cyst or symptom recurrence	6 months	Interarticular portion of L5 was fixed with a titanium laminar screw
Telfeian AE et al. [19]	51	Female	Not mentioned	Right L4-5 cyst with compression of L4 nerve	Transforaminal endoscopic operative technique for resection	No symptom recurrence	2 years	Minimally invasive, no facet bone removal
	71	Female	Not mentioned	Right L5-S1 cyst	Transforaminal endoscopic operative technique for resection	No symptom recurrence	2 years	Minimally invasive, no facet bone removal
Torres Campa-Santamarina J et al. [20]	65	Female	3-month history of sciatic pain	Left L5-S1 cyst with compression of L5 nerve	Extraforaminal tubular microscopic endoscopy-assisted approach for resection	No symptom recurrence	3 years	Minimally invasive Wiltse approach, limited facet bone damage

Three cases employed minimally invasive surgery (MIS) techniques, such as endoscopic and Wiltse approaches [19,20] (Table 1). MIS techniques can reduce blood loss and limit incidental soft tissue damage; in the context of intraforaminal LSC resection, preserving the structural integrity of the facet joint can preclude the need for fusion [20]. Due to the increased risk of complications in fusion surgeries compared to decompressive procedures alone, these endoscopic approaches may be most appropriate for patients with pre-existing facet joint stability [7,20]. In cases with significant preoperative facet instability, traditional fusion may be warranted, although more conclusive evidence is needed [6,7].

In our case report, for Case 1, fusion was warranted because the patient had degenerative spondylolisthesis, which may have caused spinal instability after surgery (Figure 1B). Other indications that necessitate fusion include neurogenic claudication, degenerative scoliosis, and radiculopathy due to foraminal stenosis. Furthermore, fusion is an established treatment modality for certain cases of trauma, infection, or neoplasia [21,22]. For Case 2, fusion was not warranted because the patient did not have significant degenerative spine changes, hence spinal instability after cyst removal was not a major concern.

## Conclusions

This report introduces two cases of unusually located, intraforaminal LSCs. One patient underwent decompressive laminectomy and cyst resection, while the other required concomitant interbody fusion. Both reported symptom resolution with no cyst recurrence at follow-up visits.

As the efficacy of fusion, decompressive laminectomy, and MIS techniques are considered, this study provides further evidence that surgical resection of LSCs can be successful, even when cysts are located in unusual places. Fusion may be warranted in some instances due to cyst location or size, spinal instability after cyst removal due to degenerative changes in the spine, or the surgical approach (minimal invasive vs. open). MIS techniques usually carry a lower risk of destabilization, and fusion may not be necessary. Further published case studies are warranted, as the restricted sample size limits the establishment of any conclusive guidelines for this uncommon condition.
